# Effects of Paternal Preconception Vapor Alcohol Exposure Paradigms on Behavioral Responses in Offspring

**DOI:** 10.3390/brainsci10090658

**Published:** 2020-09-22

**Authors:** Richa S. Rathod, Carolyn Ferguson, Amit Seth, Annalisa M. Baratta, Sonja L. Plasil, Gregg E. Homanics

**Affiliations:** 1 Department of Anesthesiology & Perioperative Medicine, University of Pittsburgh School of Medicine, Pittsburgh, PA 15261, USA; rsrathod@pitt.edu (R.S.R.); fergusonc@anes.upmc.edu (C.F.); ams649@pitt.edu (A.S.); 2 Center for Neuroscience, University of Pittsburgh School of Medicine, Pittsburgh, PA 15261, USA; amb461@pitt.edu; 3 Department of Pharmacology & Chemical Biology, University of Pittsburgh School of Medicine, Pittsburgh, PA 15261, USA; slp114@pitt.edu; 4 Department of Neurobiology, University of Pittsburgh School of Medicine, Pittsburgh, PA 15261, USA

**Keywords:** alcohol, alcohol use disorders, chronic intermittent ethanol exposure, intergenerational inheritance, paternal alcohol

## Abstract

We and others previously reported that paternal preconception chronic ethanol exposure leads to molecular, physiological, and behavioral changes in offspring including reduced ethanol consumption and preference relative to controls. The goal of the present study was to further explore the impact of paternal ethanol exposure on a wide variety of basal and drug-induced behavioral responses in first generation offspring. Adult male mice were exposed to chronic intermittent vapor ethanol or control conditions for 5–6 weeks before being mated with ethanol-naïve females to produce ethanol (E)- and control (C)-sired offspring. E-sired male offspring showed stress hyporesponsivity in a stress-induced hyperthermia assay and E-sired female offspring had reduced binge-like ethanol consumption in a drinking in the dark assay compared to C-sired offspring. E-sired offspring also showed altered sensitivity to a sedative/hypnotic dose of the GABAergic drug midazolam, but not ketamine or ethanol, in a loss of the righting response assay. E-sired offspring did not differ from controls in marble burying, novel object location, novel object recognition, social interaction, bottle-brush, novelty suppressed feeding, prepulse inhibition, every-other-day ethanol drinking, or home cage activity assays. This study adds to a growing body of literature suggesting that like in utero alcohol exposure, paternal preconception alcohol exposure can also have effects that persist and impact behavior of offspring.

## 1. Introduction

Alcohol use is an integral part of the lifestyle of many individuals and alcohol abuse is the third leading cause of preventable death in the United States [1]. According to the National Survey on Drug Use and Health, 86.4% of people aged 18 or older drank alcohol at some point in their lives and 56% reported alcohol consumption in the past month [2]. To date, only three medications are approved for alcohol dependence or prevention of relapse by the United Stated Food and Drug Administration: Disulfiram, naltrexone, and acamprosate [3,4]. Further, these medications are underprescribed and none are effective in a high percentage of patients [5].

Since a significant portion of the drinking population is of reproductive age, pre- and periconception alcohol consumption is of great importance. It is not surprising that since the fetus develops in the maternal uterine environment, the study of alcohol effects on offspring development has focused mainly on maternal contributions, and the paternal preconception environment has been largely overlooked. However, emerging evidence suggests that paternal preconception exposures to a wide variety of environmental provocations including toxicant exposure produce complex phenotypes in offspring, likely by transmission of epimutations to the next generation via sperm [6,7,8,9]. Given the high prevalence of alcohol consumption in men [10], more research in this area is warranted to understand the intergenerational effects of paternal preconception alcohol use on offspring health and behavior.

Remarkably, rodent studies have demonstrated that paternal preconception ethanol (PPE) exposure leads to molecular, cellular, physiological, and behavioral abnormalities in offspring (for reviews, see: [11,12]). For example, PPE-sired offspring exhibited insulin hypersensitivity and upregulated transforming growth factor beta signaling [13], altered enzymes and proteins involved in metabolism and weight gain [14], and altered levels of neurotrophins like brain-derived neurotrophic factor and nerve growth factor [15,16]. Paternal alcohol consumption impaired spatial learning performance in the eight-arm radial maze in male offspring while there was no change observed in an object exploration/recognition task compared to controls [17]. PPE impaired spatial memory in offspring [18], and resulted in hyperactivity and impulsivity-like behaviors [19]. Paternal ethanol drinking resulted in increased locomotor activity in an open field, and decreased sensitivity to novelty in rats [20], although our lab found no change in locomotor activity in the open field in mice [21]. Overall, in a variety of studies using different exposure paradigms and species, paternal ethanol exposure has been shown to affect a variety of molecular and behavioral phenotypes in offspring. 

A rapidly growing number of studies have demonstrated effects of PPE on ethanol drinking behavior and behavioral responses to ethanol. For example, we previously reported that ethanol-sired male offspring had reduced ethanol consumption and preference and increased sensitivity to the anxiolytic effect of a low dose of ethanol relative to controls [16,21,22]. In a conditioned place preference assay, Ceccanti et al. [15] reported that ethanol-sired offspring spent more time in a low dose, ethanol-paired compartment relative to control-sired offspring, while ethanol-sired offspring developed aversion to a high dose, ethanol-paired compartment that was rewarding to controls. Hollander et al. reported that ethanol-sired offspring had increased ethanol consumption compared to control-sired offspring [23]. Thus, studies by us and others have clearly established that paternal ethanol exposure prior to conception can have persistent effects that influence ethanol drinking and behavioral sensitivity to ethanol in the next generation. 

There are three major experimental methods of ethanol administration that have been used in in PPE studies: oral (through diet, water, or intubation), injection, and inhalation [24]. The alcohol vapor inhalation is a noninvasive model which subjects rodents to multiple cycles of intermittent ethanol exposure and withdrawal episodes. This method achieves intermittent high and sustained blood alcohol levels and has been demonstrated to induce tolerance and dependence [25,26]. Ethanol vapor exposure is also less labor intensive than other methods, it overcomes the animal’s aversion for oral ethanol administration, and multiple animals can be placed in the chamber at one time [25,27]. In the current study, two different versions of chronic intermittent ethanol (CIE) vapor exposure was employed as described below.

Although numerous studies have demonstrated intergenerational effects of paternal preconception ethanol exposure on offspring behavior, our understanding of the extent of these persistent effects of ethanol is incomplete. The present study sought to survey the effects of PPE CIE vapor exposure on tests in offspring that probe a variety of basal and drug-sensitive behaviors. Furthermore, we explored the effects of two different paternal preconception CIE paradigms on offspring behavior. The overall hypothesis tested was that paternal ethanol exposure prior to conception impacts behavioral responses in adult offspring. 

## 2. Materials and Methods

### 2.1. Animals

All experiments were approved by the Institutional Animal Care and Use Committee of the University of Pittsburgh and conducted in accordance with the National Institutes of Health Guidelines for the Care and Use of Laboratory Animals. Seven-week-old, ethanol-naïve, specific pathogen free C57BL/6J mice were purchased from the Jackson Laboratory (Bar Harbor, ME, USA). Mice were habituated to the University of Pittsburgh animal facility for at least 1 week prior to initiation of experiments. Mice were housed under 12 h light/dark cycles and had ad libitum access to food and water. 

### 2.2. Chronic Intermittent Ethanol (CIE) Inhalation

**Figure 1:**
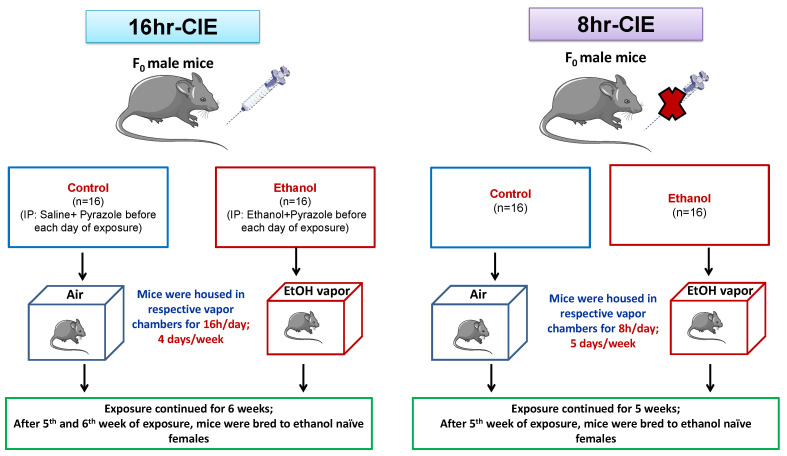
Overview of experimental design. Two experiments were carried out differing in the duration of ethanol exposure: 16 and 8 h chronic intermittent ethanol (CIE). In the 16hr-CIE experiment, before exposure initiation each day, ethanol sires were given an intraperitoneal (i.p.) injection of ethanol and pyrazole. Immediately following injection, mice were exposed to ethanol or room air control conditions in vapor chambers for 16 h/day, 4 days/week, for 6 weeks. In the 8hr-CIE paradigm, mice were placed in their respective vapor chambers for 8 h/day, 5 days/week for 5 weeks without pre-exposure injections. Immediately after the final ethanol exposure, ethanol and control-exposed males were bred in the home cage of two 8-week-old ethanol-naïve C57BL/6J female mice.

Chronic intermittent ethanol inhalation was performed as previously described [16,21,28]. Briefly, 9–10-week-old male C57BL/6J mice were randomly assigned into groups receiving either chronic intermittent vaporized ethanol (E) or room air (C). Two experiments were carried out differing in the duration of ethanol exposure (Figure 1). In the first experiment, before exposure initiation each day, ethanol sires were given an intraperitoneal (i.p.) injection of 1.5 g/kg ethanol (Decon Labs, PA, USA) and 68 mg/kg pyrazole (Sigma-Aldrich, P56607-5G), and control sires received an i.p. injection of 1.5 g/kg saline and 68 mg/kg pyrazole. Pyrazole is an alcohol dehydrogenase inhibitor that was used to maintain high blood ethanol concentrations (BECs) during ethanol vapor exposure. Immediately following injection, mice were exposed to ethanol or room air control conditions in vapor chambers for 16 h/day, 4 days/week, for 6 weeks. For the second experiment, mice were placed in their respective vapor chambers for 8 h/day, 5 days/week for 5 weeks without pre-exposure injections. For both experiments, room air was flowed into two heated Erlenmeyer flasks at a rate of 8 L/min; one flask received ethanol from a syringe pump (Harvard Apparatus, Holliston, MA, USA) while the other flask received no ethanol.

Animals were group-housed throughout the exposure. Temperature and humidity of the chambers were monitored daily at the end of each day of exposure. Body weights and rectal temperatures of mice were taken weekly. Blood ethanol concentrations were measured after the final exposure of each week by extracting tail vein blood using heparin-coated capillary tubes (Drummond, Broomall, PA, USA) and running plasma samples (extracted from blood by centrifugation at 2300× *g* for 10 min) on an Analox Alcohol Analyzer (AM1, Analox Instruments, London, UK). Ethanol content in the ethanol inhalation chamber was monitored using a custom sensor generously provided by Brian McCool (Wake Forest University), and flow rates in the chambers were adjusted weekly based on blood ethanol concentration measurements made during the preceding week. 

### 2.3. Breeding Scheme

Immediately after the final ethanol exposure, E- and C-exposed males were bred in the home cage of two 8-week-old ethanol-naïve female C57BL/6J mice for 48 h. In the 16hr-CIE exposure, offspring for cohort 1 were produced from mating after 5 weeks of CIE exposure; males were then given a 6th week of CIE exposure followed by a second round of mating to produce cohort 2. For the 8hr-CIE, all offspring were produced from one mating period after the 5th week of CIE exposure and split into two cohorts. The number and size of resulting litters sired were recorded, and offspring were weighed weekly beginning at 3 weeks of age. Weaned offspring were housed in cages consisting of two C-sired and two E-sired mice based on gender. 

### 2.4. Behavioral Experiments 

The offspring generated from both ethanol exposure paradigms were assessed in a variety of behavioral experiments. All offspring used were at least 8 weeks of age when behavioral experimentation began. Mice were housed in a room separate from the testing room. All animals were acclimated to the testing room 1 h prior to start of each assay. Behavioral experiments were performed in the light phase unless otherwise noted, and testing was done each day over a 3-h period beginning 2 h after start of light cycle. The list and sequence of all behavioral experiments performed in the offspring of both exposure paradigms are given in Table 1.

#### 2.4.1. Object Location Memory Task

The object location memory (OLM) test was performed according to a slightly modified procedure previously described [29]. Mice were habituated for one 5-min session for four days in a 17 × 17 × 12 inches open field chamber (Med Associates, Fairfax, VT, USA) with clear plexiglass walls and a white opaque floor. Then, 24 h after the last habituation session, mice underwent training sessions with two identical objects (inverted 50 mL conical tubes filled with water as weight) for a total of 10 min. A total of 30 min after the training session, subjects were placed back in the chamber for 5 min with one object displaced to a new location while the other object was not moved. All sessions were video recorded, and the time spent exploring each object was hand-scored by an observer blind to the experimental groups. Exploration time was defined as time the mouse spent with its nose within a ≈ 1 cm diameter of the object. A second observer scored a subset of the sessions to confirm that the scoring definition was reproducible. The percent preference for the displaced object was calculated as time spent exploring the displaced object relative to the total time spent exploring both objects.

#### 2.4.2. Novel Object Recognition Memory Task

The object recognition memory test was performed according to a previously described procedure [29], with minor modifications. Mice were habituated for one 5-min session daily for 4 days in the 17 × 17 × 12 inches open field chamber (Med Associates) followed by a 10-min training session on day 5 with two identical objects: inverted 50 mL conical tubes filled with water as weight or a 125 mL Erlenmeyer flask filled with black marbles for weight. Then, 30 min after the training session, animals were placed back in the chamber for a 5-min test session with one object replaced by the novel object (NO), while the other familiar object (FO) remained the same. The designation of an object as FO and NO was counterbalanced across sire-line and sex. All sessions were videotaped, and the time spent exploring each object (as defined in the OLM test) was scored by an observer blind to the experimental groups. Exploration time was defined as time the mouse spent with its nose within a ≈1 cm diameter of the object. The percent preference for the NO was calculated as time spent exploring the NO relative to the total time spent exploring both objects (preference = NO/(FO + NO) × 100).

#### 2.4.3. Social Interaction Test

Two different apparatus were used for social interaction testing: a three-chamber test was performed on offspring generated from 16hr-CIE exposure and a one-chamber test was used on offspring generated from the 8hr-CIE exposure.

Three-chamber test: This test was conducted as previously described [30], with slight modifications. The apparatus was made in-house and consisted of a 27 × 8 inch three-chamber rectangular box with clear Plexiglas sides and a white opaque floor. The two end chambers were 11 × 8 inch and the center chamber was 5 × 8 inch. There was a cut-out door on each side of the center chamber so the mice could run freely through all three sections. An inverted wire pencil-holder (4 inches at widest end × 3 inches at narrow end × 4 inches tall) was placed in the middle of each end chamber. The assay consisted of two sessions: in the first session (habituation), the test mouse was placed at the center of the middle chamber and allowed to explore all three chambers for 10 min. In the second session, an unfamiliar (stranger) mouse of the same background strain and sex was placed inside one wire cup and a novel object (in this case, a plastic figurine, 2 inches tall) was placed inside the other. The test mouse was allowed to explore all three chambers again for 10 min. The intertrial interval between sessions was 15 min. The stranger mice used in this experiment were C57 BL/6J mice from Jackson labs which had been previously habituated for 2 consecutive days in 30 min sessions under the wire containment cups before use in the test. Social interaction was defined as the amount of time the subject spent with its nose in the target zone (defined as 1 cm around the diameter of the wire holder) of each chamber in the second session. Each session was scored using SMART video tracking software (Panlab).

One-chamber test: All parameters were the same as above for this test, except that instead of a three-chamber apparatus, an open field chamber was used (see OLM), and the wire container cups with the novel object and stranger mouse were placed in opposite corners of the chamber. The stranger mice used in this assay were strain A/J from Jackson Labs and were habituated as in the three-chamber assay. Each session was scored as in the earlier test using SMART video tracking software (Panlab).

#### 2.4.4. Loss of Righting Response (LORR)

Mice were administered ethanol (3.5 g/kg), ketamine (150 mg/kg), or midazolam (45 mg/kg) intraperitoneally. Mice were supine positioned on V-shaped plastic troughs as soon as they became ataxic. Body temperature was maintained at 35–37 °C with heat lamps. Duration of the loss of righting response (LORR) was defined as the time started from being placed in the supine position until they were able to voluntarily regain their reflexes three times within 30 s.

#### 2.4.5. Acute Functional Tolerance Test (AFT)

An acute functional tolerance (AFT) test was conducted as previously described [31] with minor modifications. Mice were trained to stay on a nonrotating 1-inch diameter rotarod (Ugo Basile, Stoelting Co., Wood Dale, IL, USA) for 1 min. Then, mice were injected with 1.75 g/kg, i.p. ethanol and they were placed back on the rotarod, and the latency to fall was recorded. Mice were tested at every 5-min until they were able to remain on the rotarod for 1 min. Tail nick blood sample was collected as soon as they were able to stay for 1 min and time was recorded (T1). Immediately, second injection of ethanol (2 g/kg, i.p.) was given and mice were tested in 10-min intervals until they were able to balance themselves for 1 min (T2) at which time a second tail vein blood sample was collected. The collected blood samples were used for measurement of BECs. AFT was calculated as the difference in BEC collected at T2 versus T1.

#### 2.4.6. Stress-Induced Hyperthermia (SIH)

Sensitivity to acute stress was evaluated by monitoring the change in body temperature associated with taking repeated rectal measurements [32]. Mice were individually housed for 1 week prior to the start of the experiment. In this procedure, body temperature was measured twice (10 min apart) using a glycerol-lubricated thermistor rectal probe (Physitemp, #HET-3). In between readings, animals were returned to their individual cages. Due to the stress experienced during the first temperature (T1) measurement, body temperature increases when measured a second time (T2). This difference in temperature (ΔT = T2 − T1) is defined as the stress-induced hyperthermia (SIH) response.

#### 2.4.7. Drinking in the Dark (DID) Assay 

Single-housed mice were offered 20% ethanol (*w*/*v*) from a sipper tube made from a 10 mL pipet for 2 h on the first day followed by a 4-h period on the second day. Each test period started 3 h after the start of the dark cycle. The volume of ethanol solution was recorded before and after each drinking session, and ethanol consumption was calculated (g ethanol consumed/kg body weight). At the end of the 4-h session, a blood sample was collected from a tail nick to determine the blood ethanol concentrations.

#### 2.4.8. Every-Other-Day Two-Bottle Choice (EOD 2BC) Assay

Mice were single-housed and acclimated to drinking from two sipper tubes made from 25 mL pipets containing only water for 1 week. Subsequently, mice were introduced to every-other-day two-bottle choice (EOD 2BC) ethanol drinking by offering escalating concentrations of 3%, 6%, and 10% ethanol (*w*/*v*) for 24 h in one of the two drinking tubes on Monday, Wednesday, and Friday, respectively, during the first week. During the following 3 weeks, 20% ethanol (*w*/*v*) was provided in one of the drinking tubes for 24-h sessions on Monday, Wednesday, and Friday. The position of the water and ethanol sipper tubes were switched during each test day to avoid side preference.

### 2.5. Statistical Analysis

Behavioral experiments were analyzed using Student’s *t*-test and two-way ANOVAs with or without repeated measures where appropriate. For ANOVA results reaching statistical significance (*p* < 0.05), post-hoc pairwise comparisons were made using Bonferroni multiple comparison’s test. In midazolam-induced LORR assay for males in 16hr-CIE experiment, two outliers were removed as determined by the Rout test. For EOD 2BC drinking assay, two-way ANOVA with mixed effect analysis was used. In all behavioral assays, males and females were tested on different days; therefore, male and female test results were analyzed separately.

## 3. Results

### 3.1. Paternal Preconception CIE Exposure

**Figure 2:**
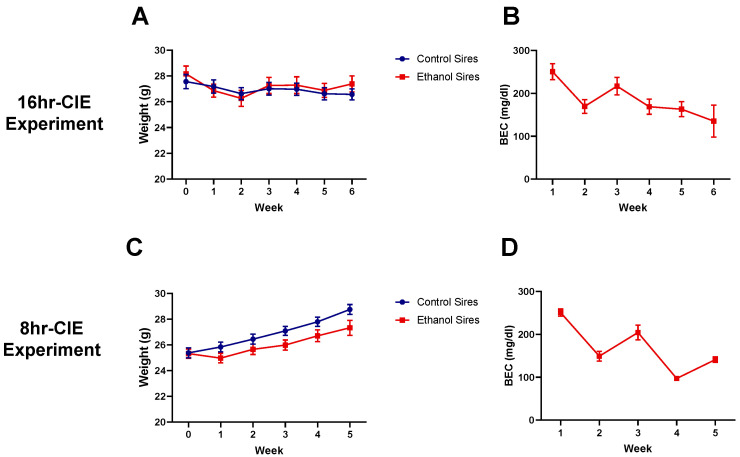
Paternal preconception CIE exposure. (**A**) From the 16hr-CIE exposure paradigm, there were no differences in body weights between treatment groups and neither group gained weight during the exposure period. (**B**) Mean blood ethanol concentrations (BECs) for 16hr-CIE sires. (**C**) From the 8hr-CIE exposure paradigm, both treatment groups gained weight over the duration of the exposure with a significant main effect of time and treatment. (**D**) Mean BECs for 8hr-CIE sires. Data presented as mean ± SEM (*n* = 16).

Adult male C57BL/6J mice were exposed to CIE or room air conditions for 5 or 6 weeks prior to mating. Two different exposure experiments were carried out: 16hr-CIE (16 h/day, 4 days/week, with daily pyrazole + ethanol injections; sires were mated after weeks 5 and 6) and 8hr-CIE (8 h/day, 5 days/week, no injections; sires were mated after week 5). Notably, in 16hr-CIE experiment, the sires in both treatment groups did not gain weight throughout the exposure period. There was a significant effect of time × sire interaction (F (4, 120) = 2.70, *p* < 0.05; Figure 2A) on body weights, but post-hoc analysis revealed no significant difference between treatment groups for any of the weeks. In contrast to the 16hr-CIE, the 8hr-CIE sires in both treatment groups gained weight over the duration of the exposure. Further, there was no time × sire interaction but a significant main effect of time (F (4, 120) = 36.0, *p* < 0.0001) and a small but significant main effect of treatment (F (1, 30) = 4.47, *p* = 0.043; Figure 2C) on sire body weight. The average blood ethanol concentrations across all weeks of paternal 16hr-CIE was 193.8 ± 17.2 mg/dL (Figure 2B) and 8hr-CIE was 168.5 ± 26.7 mg/dL (Figure 2D).

### 3.2. Breeding Results

Immediately following the final exposure, each sire was caged with two ethanol-naïve C57BL/6J females for 48 h. The 16hr-CIE experiment was conducted first. Fewer ethanol-exposed males (50%) sired litters than did control males (75%), although the difference was not significant. The number of ethanol-exposed males producing litters with viable offspring was significantly less than control males in this experiment (Fisher’s exact, *p* < 0.05; see Table 2). 

In the 8hr-CIE, there was a higher percent of males from both treatments who sired litters compared to the 16hr-CIE, and a higher percent of males in both treatments siring viable litters compared to the 16hr-CIE. A lower percent of ethanol-sires had viable offspring compared to control sires, but the difference was not significant. Average litter sizes of all litters producing living offspring were not affected by sire treatment and were comparable across exposures.

### 3.3. Body Weights of Offspring

**Figure 3:**
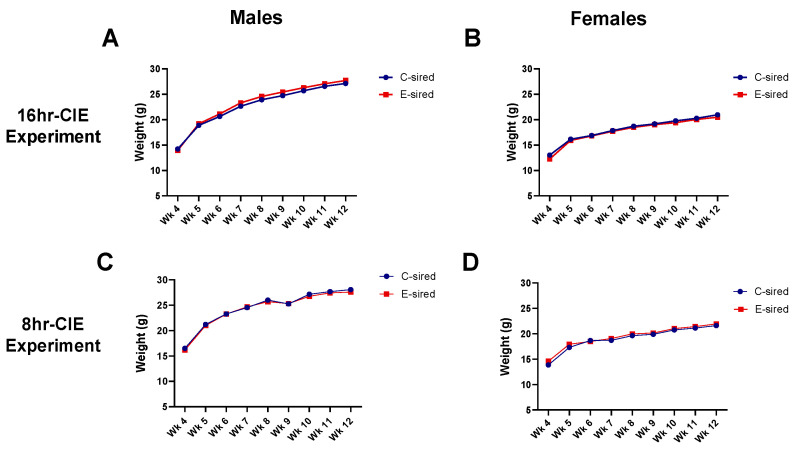
Paternal preconception CIE exposure did not affect the body weights of offspring between treatment groups. (**A**) 16hr-CIE male offspring body weight. (**B**) 16hr-CIE female offspring body weight. (**C**) 8hr-CIE male offspring body weight. (**D**) 8hr-CIE female offspring body weight. Data presented as mean ± SEM (*n* = 30–34). WK: weeks.

The body weights of offspring were recorded from weaning through 3 months of age (Figure 3). In the 16hr-CIE experiment for body weight of male offspring, there was a significant main effect of time (F (2.037, 134.4) = 1652, *p* < 0.001) and time × treatment interaction (F (8, 528) = 2.092, *p* < 0.05), but no effect of treatment (Figure 3A). For body weight of female offspring, there was a significant main effect of time (F (2.764, 174.1) = 1084, *p* < 0.001) and time × treatment interaction (F (8, 504) = 1.968, *p* < 0.05), but no effect of treatment (Figure 3B). In the 8hr-CIE experiment for body weight of male offspring, there was a significant main effect of time (F (7, 418) = 649.3, *p* < 0.001) but no effect of treatment or treatment × time interaction (Figure 3C). Similarly, for body weight of female offspring, there was a significant effect of time (F (8, 460) = 417.6, *p* < 0.001) but no effect of treatment or interaction between treatment and time (Figure 3D).

### 3.4. Behavioral Assay Results of Adult Offspring

#### 3.4.1. Object Location Memory Test (OLM)

**Figure 4:**
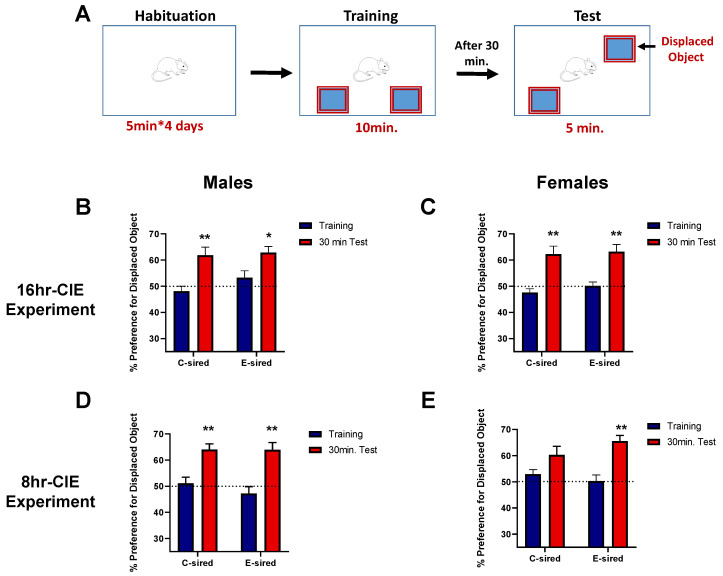
Effects of paternal preconception ethanol exposure on the object location memory test in offspring. (**A**) Diagrammatic depiction of the experimental set-up for the object location memory test. In the 16hr-CIE experiment, both C- and E-sired (**B**) male and (**C**) female progeny showed a preference for the displaced object 30 min after training. Similarly, in the 8hr-CIE experiment, (**D**) C- and E-sired male, and (**E**) E-sired female offspring showed a preference for the displaced object 30 min after training. Percent preference was calculated as (time exploring displaced object/total time exploring both objects) × 100. For all panels, * *p* < 0.05; ** *p* < 0.001 comparing training versus test, Bonferroni post-hoc test. Data presented as mean ± SEM (*n* = 15–20 mice per sex per treatment group).

OLM is most commonly used to assess cognition, specifically spatial memory and discrimination, in rodents (Figure 4A). In the 16hr-CIE experiment, C- and E-sired offspring spent equal time exploring the objects during the training session (Figure 4B,C). There was a significant effect of session (training vs. test) in both male (F (1, 34) = 23.88, *p* < 0.001; Figure 4B) and female offspring (F (1, 38) = 33.63, *p* < 0.001; Figure 4C) but no effect of treatment or interaction between treatment and session on percent preference for displaced object. Bonferroni post-hoc testing revealed that both C- and E-sired offspring showed a preference (*p* < 0.001) for the displaced object during the test session as compared to the training session, indicating that object location memory was not influenced by paternal preconception ethanol exposure.

Similarly, in the 8hr-CIE experiment, C- and E-sired offspring spent equal time exploring the objects during training (Figure 4D,E). There was a significant effect of session in both male (F (1, 29) = 39.44, *p* < 0.001; Figure 4D) and female offspring (F (1, 30) = 24.00, *p* < 0.001; Figure 4E) but no effect of treatment or interaction between treatment and session on percent preference for displaced object. Bonferroni post-hoc testing revealed that both C- and E-sired male and female offspring showed a preference (*p* < 0.001) for the displaced object during the test session. While preference for the displaced object did not reach significance in C-sired female offspring, there was a trend (*p* = 0.06) when comparing the training and testing sessions.

#### 3.4.2. Object Recognition Memory (ORM)

**Figure 5:**
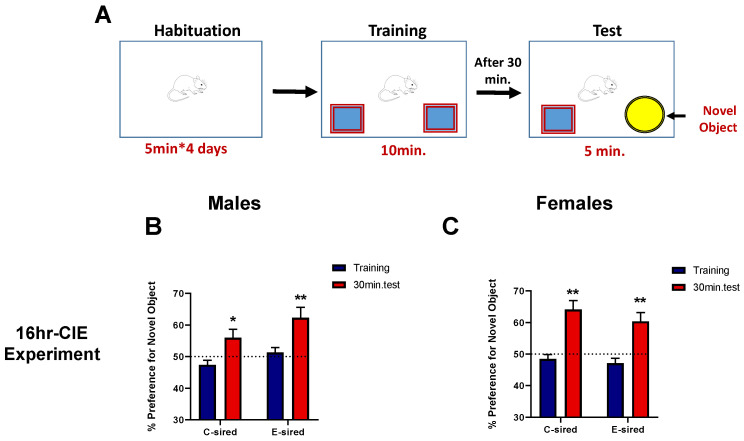
Effects of paternal preconception ethanol exposure (16hr-CIE experiment) on the object recognition memory in offspring. (**A**) Overview of the object recognition memory paradigm employed. (**B**) Male and (**C**) female progeny showed a preference for the novel object 30 min after training. Percent preference was calculated as (time spent exploring novel object/total time exploring both objects) × 100. For all panels, * *p* < 0.05; ** *p* < 0.001 comparing training versus test, Bonferroni post-hoc test. Data presented as mean ± SEM (*n* = 15–20 mice per sex per treatment group).

Object recognition memory (ORM) was used to investigate learning and memory in mice by evaluating the differences in the exploration time of familiar and novel objects (Figure 5A). This test was performed only on offspring generated from the paternal 16hr-CIE exposure paradigm. A significant effect of treatment (F (1, 26) = 4.490; *p* < 0.05) and session (F (1, 26) = 17.24; *p* < 0.001) but no interaction on percent preference for novel object was observed in male offspring. Post-hoc analysis revealed that both C- and E-sired male offspring showed a preference for the novel object in the test session over training session (*p* < 0.05), while no effect of treatment was observed by session (Figure 5B). Similarly, in female offspring, there was a significant effect of session (F (1, 30) = 50.81, *p* < 0.001; Figure 5C), but no effect of treatment or treatment and session interaction. Post-hoc analysis revealed that both C- and E-sired female offspring showed a preference (*p* < 0.05) for novel object during the test session compared to the training session suggesting no difference between object recognition memory between C- and E-sired offspring.

#### 3.4.3. Social Interaction Test

**Figure 6:**
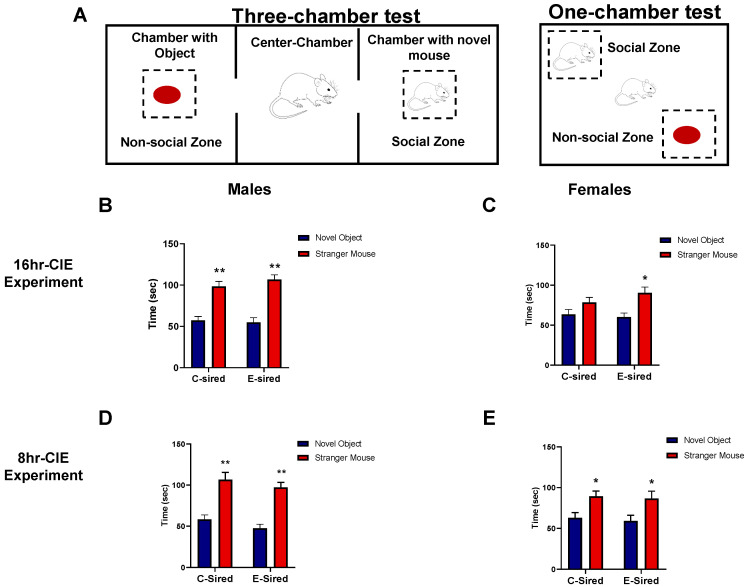
Effects of paternal preconception ethanol exposure on social interaction test in offspring. (**A**) Diagrammatic depiction of the experimental set-up for the social interaction test. In the 16hr-CIE experiment, both C- and E-sired (**B**) male and (**C**) E-sired female progeny showed a preference for the stranger mouse over a novel object. Similarly, in the 8hr-CIE experiment, C- and E-sired (**D**) male and (**E**) female offspring showed a preference for the stranger mouse over the novel object. For all panels, * *p* < 0.05; ** *p* < 0.001 comparing novel object versus stranger mouse, Bonferroni post-hoc test. Data presented as mean ± SEM (*n* = 15–20 mice per sex per treatment group).

The social interaction test measures the level of sociability by comparing the time a mouse spends in a zone with a stranger mouse (social zone) to the time in a zone with novel object present (nonsocial zone) (see Figure 6A for experimental scheme). The three-chambered social interaction test was carried out on offspring from the 16hr-CIE experiment. There was a significant effect of zone (nonsocial vs. social) in both male (F (1, 32) = 64.92, *p* < 0.001, Figure 6B) and female (F (1, 36) = 10.26, *p* < 0.001, Figure 6C) offspring but no effect of treatment or interaction between treatment and zone on time spent exploring stranger mouse and object. Post-hoc analysis revealed that both C- and E-sired male (*p* < 0.001) and E-sired female (*p* < 0.05) offspring spent more time with the novel mouse as compared to novel object.

The one-chamber social interaction test was carried out on offspring sired from the 8hr-CIE exposure paradigm. Similar results were observed to those of the offspring sired from 16hr-CIE experiment. There was a significant effect of zone (nonsocial vs. social) in both male (F (1, 58) = 57.69, *p* < 0.001; Figure 6D) and female (F (1, 60) = 13.48, *p* < 0.001; Figure 6E) offspring but no effect of treatment or interaction between treatment and zone on time spent exploring stranger mouse and object. Post-hoc analysis revealed that both C- and E-sired male (*p* < 0.001) and female (*p* < 0.05) offspring spent more time with the novel mouse as compared to the novel object.

#### 3.4.4. Loss of Righting Response (LORR)

**Figure 7:**
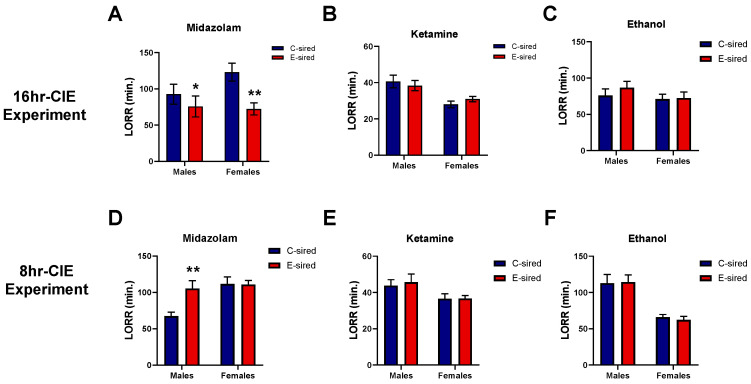
Effects of paternal preconception ethanol exposure on drug sensitivity in offspring. In the 16hr-CIE exposure, (**A**) E-sired male and female progeny showed shorter LORR duration compared to control offspring, while (**B**) ketamine and (**C**) ethanol-induced LORR did not differ between treatment groups. In 8hr-CIE exposure, (**D**) E-sired male offspring showed longer LORR duration while no change observed in female offspring. (**E**) Ketamine and (**F**) ethanol-induced LORR did not differ between treatment groups. For all panels, * *p* < 0.05; ** *p* < 0.001 comparing C-sired versus E-sired, Bonferroni post-hoc test. Data presented as mean ± SEM (*n* = 12–16 mice per sex per treatment group).

We tested the sensitivity of offspring to the sedative/hypnotic effects of midazolam, ketamine, and ethanol using a LORR assay. Midazolam is a popular sedative and exhibits its hypnotic or anesthetic actions by binding to gamma-aminobutyric acid-A (GABAA) receptors [33]. In the 16hr-CIE experiment, midazolam LORR duration was shorter in E-sired male (t25 = 2.219, *p* < 0.05) and female (t21 = 3.126, *p* < 0.01) offspring as compared to same sex C-sired offspring (Figure 7A). Conversely, in the 8hr-CIE experiment, midazolam LORR duration was longer in E-sired male offspring (t28 = 3.309, *p* < 0.001) as compared to C-sired male offspring while there was no difference observed in female offspring (Figure 7D). The duration of ketamine (Figure 7B,E) and ethanol (Figure 7C,F) induced LORR did not differ between C- and E-sired offspring generated from either experimental paradigm. 

#### 3.4.5. Acute Functional Tolerance Test (AFT)

**Figure 8:**
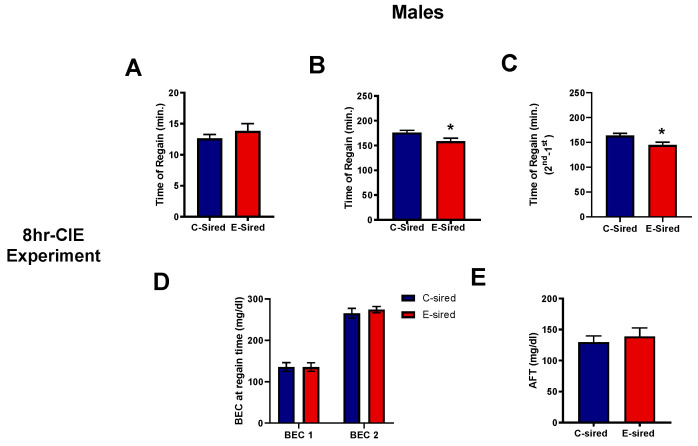
Effects of paternal preconception ethanol exposure (8hr-CIE) on acute functional tolerance to ethanol in male offspring. (**A**) Time needed to regain the ability to remain on the rotarod for 60 s after 1st injection and (**B**) 2nd injection. (**C**) Differences in the time to regain balance between the 2nd and 1st ethanol injections, and (**D**) BECs (mg/dL) measured at the time of regained balance after each ethanol injection. (**E**) Acute functional tolerance (AFT) measured as the differences in BECs after each ethanol injection. * *p* < 0.05; comparing C-sired versus E-sired, *t*-test. Data presented as mean ± SEM (*n* = 14–16 mice per sex per treatment group).

This test was performed on the offspring generated from the 8hr-CIE experiment. The time to recover after the first ethanol injection was not significantly altered in E-sired offspring compared to C-sired offspring (Figure 8A). After the second ethanol injection, recovery time was significantly shorter in E-sired male offspring as compared to C-sired male offspring (t27 = 2.25, *p* < 0.05; Figure 8B). The time of regain between two injections was significantly shorter (t27 = 2.55, *p* < 0.05; Figure 8C) in E-sired male offspring compared to controls. However, BEC measurements (Figure 8D) and AFT (Figure 8E) were not significantly altered between treatment groups. There was no significant change in female offspring as a result of paternal ethanol exposure in any of the parameters discussed above.

#### 3.4.6. Stress-Induced Hyperthermia (SIH)

**Figure 9:**
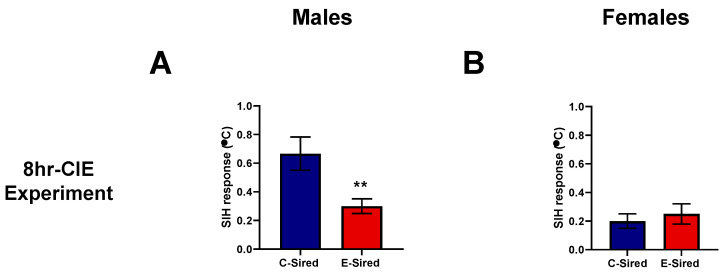
Effects of paternal preconception ethanol exposure (8hr-CIE) on stress-induced hyperthermia response (SIH) in offspring. SIH response in (**A**) male and (**B**) female offspring. ** *p* < 0.01; comparing C-sired versus E-sired, *t*-test. Data presented as mean ± SEM (males, *n* = 14; females, *n* = 11).

SIH was used to measure the stress-induced response in offspring generated from the 8hr-CIE experiment. E-sired males showed a reduced SIH response as compared to C-sired males (t27 = 2.808, *p* < 0.01; Figure 9A) while female offspring SIH response was not influenced by paternal preconception ethanol exposure (Figure 9B).

#### 3.4.7. Drinking in the Dark (DID)

**Figure 10:**
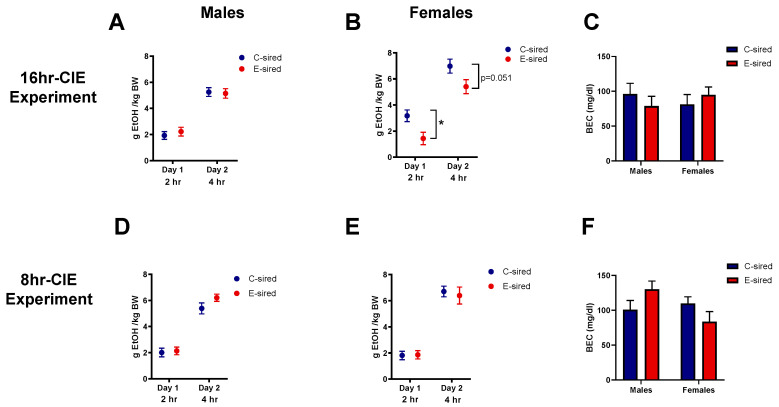
Effects of paternal preconception ethanol exposure on offspring ethanol consumption in drinking in the dark assay. For the 16hr-CIE experiment, ethanol consumption in (**A**) males was unaltered while (**B**) reduced in females. (**C**) BECs measured at the end of the 2nd session in offspring were similar between treatment groups. For the 8hr-CIE experiment, ethanol consumption in (**D**) males (**E**) females was similar between treatment groups. (**F**) BECs measured at the end of the second session in offspring were not altered between treatment groups. * *p* < 0.05; comparing C-sired versus E-sired, *t*-test. Data presented as mean ± SEM (males, *n* = 14; females, *n* = 11–13).

The drinking in the dark (DID) assay is used to measure binge-like ethanol drinking patterns in rodents. E-sired male offspring showed no significant differences from C-sired for ethanol consumption in either experiment (Figure 10A,D). However, E-sired females had significantly reduced ethanol consumption on the first day (t22 = 2.639, *p* < 0.05) with a trend towards reduction on the second day (t22 = 2.065, *p* = 0.0509) compared to C-sired females (Figure 10B) in the 16hr-CIE experiment while no change was observed in the 8hr-CIE experiment (Figure 10E). BECs of samples collected at the end of a 4 h drinking session on day 2 were similar between C- and E-sired offspring in both experiments (Figure 10C,F).

#### 3.4.8. EOD 2BC Drinking Assay

**Figure 11:**
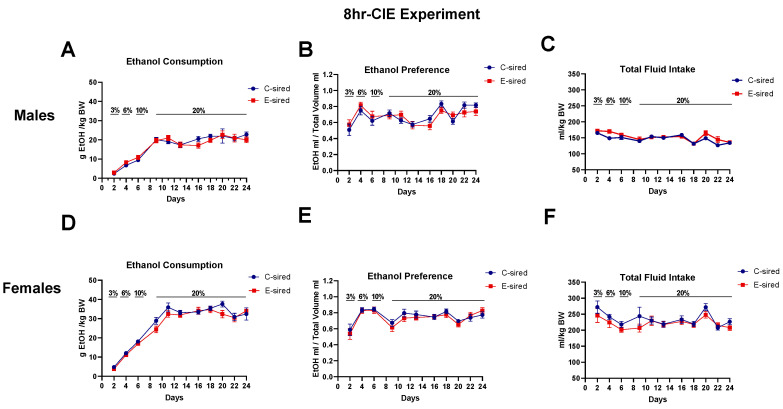
Paternal preconception ethanol exposure (8hr-CIE) did not alter offspring ethanol consumption in every-other-day two-bottle choice (EOD 2BC) assay. (**A**) Ethanol consumption in males. (**B**) Ethanol preference in males. (**C**) Total volume of fluid consumed by males. (**D**) Ethanol consumption in females. (**E**) Ethanol preference in females. (**F**) Total volume of fluid consumed by females. Data presented as mean ± SEM (*n* = 16 per group).

This assay is a model of rapid escalation of ethanol drinking in mice. Ethanol consumption and preference were measured in offspring generated from the 8hr-CIE experiment. There was a significant main effect of days on ethanol consumption (F (3.6, 103.6) = 64.10, *p* < 0.01, Figure 11A) and ethanol preference (F (10, 165) = 6.49, *p* < 0.01, Figure 11B) but no effect of sire treatment or days and sire treatment interaction. Further, there was a main effect of days (F (6.572, 182.1) = 13.53, *p* < 0.01; Figure 11C) and sire treatment and days interaction (F (10,277) = 1.9, *p* < 0.05) on total fluid consumption in male offspring, but no effect of sire treatment. In females, there was a significant main effect of days on ethanol consumption (F (5.16, 146.2) = 113.3, *p* < 0.001; Figure 11D), ethanol preference (F (10, 150) = 8.3, *p* < 0.001; Figure 11E), and total fluid consumption (F (3.4, 96.69) = 4.822, *p* < 0.01; Figure 11F) but no effect of sire treatment or interaction between variables observed. Post-hoc analysis did not show any significant differences between C-sired and E-sired female offspring on ethanol consumption, preference, and total fluid consumption.

#### 3.4.9. Additional Behavioral Results

Several other behavioral assays were performed including marble burying test (test of anxiety-like behavior) (Figure S1), novelty suppressed feeding test (test for stress-induced anxiety; Figure S2), home cage activity (to monitor locomotor activity in home cage; Figure S3), prepulse inhibition test (measure of sensorimotor gating; Figure S4), bottle-brush test (used to reveal irritability-like behavior; Figure S5), and blood glucose levels (blood glucose is an essential parameter in the study of metabolism and diabetes; Figure S6). There were no significant differences observed between C- and E-sired offspring in any of these assays (see Supplementary Materials).

## 4. Discussion

In this study, we investigated the intergenerational effects of two chronic PPE paradigms on a wide variety of behavioral responses in adult offspring (see Table 3 for a summary of results). Consistent with previous studies [15,21,33] which reported that paternal ethanol exposure selectively impacted male offspring, we found that E-sired male offspring had a reduced sensitivity to stress-induced hyperthermia compared to C-sired male offspring while female E-sired offspring response did not differ from the female C-sired response on this assay. We also observed that E-sired offspring had altered sensitivity to the sedative/hypnotic effects of midazolam (but not ketamine or ethanol) that differed in a sex and paternal exposure paradigm specific manner. In a DID assay of ethanol consumption, E-sired female offspring consumed less ethanol than controls, although in the every-other-day two-bottle free choice drinking paradigm, E-sired male and female offspring ethanol consumption did not differ from controls. Additionally, PPE did not impact offspring of either sex on many behavioral responses that span diverse behavioral domains. These additional assays include marble burying, novel object location, novel object recognition, social interaction, bottle-brush, novelty suppressed feeding, prepulse inhibition, and home cage activity. This study highlights the growing appreciation that ethanol can selectively exert effects that persist across generations. These intergenerational effects are selective for specific behavioral responses, and are often sex specific.

Previous research from our laboratory has demonstrated that paternal ethanol exposure imparted stress hyporesponsivity in a stress-evoked ethanol-drinking assay and an acute restraint stress challenge selectively to male offspring [28]. In the present study, we extended these results on stress reactivity using the SIH assay. Consistent with our previous report, we found that E-sired males were hyporesponsive to SIH compared to C-sired males, but there was no difference observed in females between treatment groups. However, a caveat should be noted that control females did not show any SIH response in our study and it might be in part due to the fact that in C57BL/6 female mice, core body temperature is higher than males and known to be influenced by estrous cycle [34]. Together, these results establish that paternal ethanol exposure results in stress hyporesponsivity in males. Since stress has a major impact on overall health and well-being and can directly contribute to the development of numerous mental health disorders including drug and alcohol abuse, further research is warranted on this persistent intergenerational effect of ethanol.

We previously employed an 8hr-CIE paradigm to investigate how paternal preconception ethanol exposure impacts ethanol drinking and ethanol related phenotypes in offspring in several publications [16,21,28,35]. In the current study, we used this same 8hr-CIE paradigm and also a 16hr-CIE paradigm that is more widely used by others in the alcohol field. This second paradigm is based on that developed by Becker and colleagues and includes multiple cycles of 16 h of exposure followed by 8 h of abstinence, with each exposure cycle starting with a priming injection of ethanol and pyrazole. Pyrazole is a competitive alcohol dehydrogenase inhibitor and is used to maintain stable BECs during the 16 h exposure period. In contrast, it has been established that pyrazole is not needed to maintain high BECs during 8 h of ethanol exposure [27] and this was the determinant factor in our use of the 8hr-CIE model in our previous studies. However, the 16hr-CIE paradigm has been successfully used to produce alcohol dependence and tolerance in animal models [36,37], and importantly, the multiple cycles of 16hr-CIE cause escalation in ethanol drinking behavior [38,39].

Although the weekly BECs of the sires were similar between the two CIE paradigms, we noticed several adverse effects associated with the 16hr-CIE paradigm. Whereas control and ethanol exposed sires in the 8hr-CIE experiment gained weight across the 5-week exposure period, neither control nor ethanol exposed sires in the 16hr-CIE experiment did. This adverse effect is likely due to pyrazole toxicity since both the control and the ethanol exposed sires in the 16hr-CIE experiment received pyrazole and both failed to gain weight. Pyrazole has previously been shown to have adverse effects on body weight [40]. Secondly, reproductive performance of ethanol treated males in the 16hr-CIE experiment appeared to be reduced compared to 16 h control sires and 8 h control and ethanol sires. This was evident in the number of males siring litters and in the number of sires that produced viable offspring. This adverse effect is likely not due to pyrazole, but instead due to toxic effects of long-term exposure to high concentrations of ethanol and or confound of withdrawal from the intoxicating effects of ethanol. Several studies by others have reported adverse effects of chronic ethanol consumption on sperm quality, reproductive hormones, fertility, and reproductive performance [41,42,43,44]. 

The present study assessed sensitivity to the sedative-hypnotic effects of ethanol, ketamine, and midazolam in offspring following paternal ethanol exposure. No change in sensitivity to ethanol and ketamine was observed in either exposure paradigm. Earlier studies have reported altered ethanol-induced LORR duration in offspring in response to gestational ethanol exposure [45] while there are no studies examining such effects in response to vapor PPE that we are aware of. Further, while altered sensitivity to midazolam was observed in both exposure paradigms, the results surprisingly differed in directionality. Male and female offspring of sires from the 16hr-CIE experiment had shorter LORR duration. In contrast, in 8hr-CIE experiment, male offspring had longer LORR duration than controls. Response in females was unaltered. Midazolam is a benzodiazepine and acts as a positive allosteric modulator of GABAA receptors. Studies have demonstrated that ethanol can induce changes in GABAA receptor subunit composition and influence the agonist pharmacology of GABAA receptors depending on the concentrations of ethanol [46,47,48]. It is conceivable that subtle differences in paternal ethanol exposure paradigms resulted in differential changes in the GABAA receptor subunit composition in the ethanol-exposed males, and those alterations were transferred epigenetically to offspring resulting in the observed differential sensitivity to midazolam. However, further studies are required to identify possible mechanisms underlying dose-dependent differential sensitivity to midazolam.

We were motivated to investigate the effects of PPE on cognitive performance in offspring by prior publications which suggested that children of alcoholic fathers had impaired cognitive and academic performance compared to children whose fathers did not have an alcohol use disorder [49,50]. Furthermore, paternal preconception cocaine exposure has been demonstrated to selectively impair object place recognition, but not novel object recognition, selectively in male offspring [51].

The present study revealed that PPE in either the 16hr-CIE or 8hr-CIE experiment did not affect spatial learning and memory of offspring. Both groups of mice displayed robust learning in the object location and novel object recognition tasks by showing a preference for the displaced and novel object, respectively. Consistent with our results, previous research found that paternal ethanol consumption in rats did not affect their offspring’s object exploration/recognition memory but did show cognitive deficits during the eight-arm radial maze and T-maze performance [17]. Furthermore, cognitive alterations in T-maze performance were also reported in the offspring of male rats who were exposed to a binge-like ethanol drinking paradigm [23]. It is possible that paternal ethanol exposure may not affect object-discrimination associated spatial memory while selectively influencing other learning tasks in the offspring. Thus, it will be of further interest to explore mechanisms underlying differential responses for spatial learning and memory as a result of PPE.

Emerging evidence indicates that impaired social behaviors and social anxiety in offspring are associated with maternal alcohol use and prenatal exposure [52,53,54]. However, the effects of paternal preconception alcohol consumption on social behaviors in offspring has been relatively less explored. In this study, we assessed social approach behavior using the social interaction test to evaluate the effects of paternal ethanol exposure on offspring behavior. We found that all offspring, regardless of paternal ethanol exposure treatment, showed normal social behavior by preferring a social stimulus over an object. Recently, one study reported that paternal binge-like ethanol consumption had no effects on sociability in offspring [55], while another study showed antisocial behavior such as aggression in alcohol-sired male offspring [56]. Therefore, we assessed aggressive and defensive responses in offspring using the bottle-brush test, wherein there were no differences from controls observed in E-sired offspring. It has been suggested that alcohol use may result in either enhanced social behavior or social inhibition depending on the alcohol dose and particular exposure paradigm [57]. Thus, it is important to better understand the relationship between varied PPE paradigms and their effects on a wide range of social behaviors in subsequent generations.

The intergenerational effects of ethanol on behavioral sensitivity to ethanol and ethanol drinking behavior are of great interest as these effects could impact risk for developing an alcohol use disorder. Using the LORR assay, we found that sensitivity to the acute sedative/hypnotic effects of an acute high dose ethanol injection was unaltered by paternal ethanol exposure. We also investigated tolerance to repeated injections of ethanol using an AFT assay. While we observed that E-sired male offspring showed faster recovery from the motor ataxic effects of ethanol after the second ethanol injection, there was no change in AFT or BEC. This observation that E-sired male offspring recovered at an earlier time point, but at the same BEC as controls, suggests altered ethanol metabolism although this was not directly assessed in the current study. However, we previously observed no change in ethanol metabolism or clearance in offspring of sires exposed to the same 8hr-CIE paradigm [16]. We also tested offspring for ethanol consumption using two different ethanol drinking assays. Using the DID assay which models binge-like consumption, E-sired females had reduced ethanol consumption on day 1 and a trend towards a reduction on day 2 compared to C-sired females in the 16 h CIE exposure experiment. However, BECs were similar which suggests differences in the timing of ethanol consumption relative to sample collection. We did not observe any differences in the DID assay in offspring from 8 h CIE exposure. A recent study from our lab found reduced ethanol consumption, but only in ethanol-sired male offspring during the DID assay following a paternal voluntary alcohol drinking exposure model [22]. We also tested ethanol drinking using an every-other-day two-bottle choice drinking assay. This intermittent administration protocol has been shown to induce rapid escalation of alcohol drinking [58]. We found no difference between C- and E-sired offspring in consumption, preference, or total volume consumed. In our previous studies, male offspring of E-sired mice showed reduced consumption and preference at lower concentrations of ethanol (3%–15%) in a continuous 2BC assay [16,21]. Others have reported that paternal binge ethanol consumption results in more ethanol intake in offspring [23]. Collectively, these studies reveal that PPE can have persistent effects that influence offspring sensitivity to ethanol and ethanol drinking behavior. These effects appear to be dependent on the method of PPE exposure and effects can be sex specific. Clearly, more work needs to be conducted to more completely understand these complex effects and how they contribute to development of alcohol use disorder.

A fundamental question that arises is how do paternal experiences with ethanol persist across generations and lead to behavioral phenotypic outcomes in offspring? Growing evidence suggests epigenetic changes in sperm as the chief mechanism through which paternal effects are transmitted across generations [59,60,61]. Numerous recent studies have definitively established that epigenetic regulatory RNAs in sperm are sufficient to recapitulate the effects of environmental perturbations that are passed on following natural mating. For example, the intergenerational effects of stress [59,62], high fat diet [60], environmental enrichment [61], and trauma [63,64] are all mediated by changes in sperm noncoding RNAs. Several studies have reported that alcohol can act as an epimutagen and has the ability to induce alterations in DNA methylation, chromatin modifications, and noncoding RNAs in sperm (for review, see: [11]). Previous studies from our laboratory [35] and others [65] found that chronic ethanol exposure altered several small noncoding RNAs including tRNA derived fragments, PIWI-interacting RNAs, and miRNAs in sperm. Studies demonstrating a causal role of these alcohol-induced sperm RNA changes are desperately needed.

In conclusion, it is becoming quite evident that like many other environmental insults, paternal alcohol exposure prior to procreating can have long lived effects that can be passed through the male germline and ultimately impact behavior of offspring. Effects are wide ranging and impact a variety of behavioral domains that include alcohol drinking behavior and behavioral sensitivity to stress and alcohol. Further studies are needed to more completely characterize the impact of the intergenerational effects of ethanol.

## Figures and Tables

**Table 1:** List and sequence of all assays performed on the offspring of CIE-exposed sires.

16hr-CIE	8hr-CIE
Housing Room: Standard 7am ON-7pm OFF light cycle	
Cohort 1	Cohort 1
Marble Burying Test **Object Location Test** **Social Interaction Test** Object Recognition Test Blood Glucose Levels **LORR (Ethanol)**	**Object Location Test** **Social Interaction Test** Acute Functional Tolerance Test **LORR (Ketamine, Midazolam, and Ethanol)**
Housing Room: Reversed 10am OFF-10pm ON light cycle	
Cohort 2	Cohort 2: Single-housed
Home Cage Activity Prepulse Inhibition Test Novelty Suppressed Feeding Test **LORR (Ketamine and Midazolam)** **Drinking in the Dark**	Stress-Induced Hyperthermia Bottle-Brush Test **Drinking in the Dark** EOD/2 bottle choice

Bolded items indicate assays performed on offspring from both exposure paradigms.

**Table 2:** Breeding data for control and ethanol sires following 5 weeks of exposure.

	16hr CIE		8hr CIE **	
Parameter	C-Sired	E-Sired	C-Sired	E-Sired
Number of males exposed	16	16	15	14
Number of males siring litters	12 (75%)	8 (50%)	14 (93%)	12 (86%)
Number of sires with viable offspring	11 (69%)	4 (25%) *	12 (80%)	8 (57%)
Average litter size of viable litters	6.7	7.2	6.5	6.8

* *p* < 0.05, as compared to C-sired in 16hr-CIE exposure, ** Some litters were collected prepartum for a separate study after the 8hr-CIE exposure. Therefore, one control sire and two ethanol sires are removed from the number of males exposed in this table.

**Table 3:** Summary of behavioral assay results in adult F1 offspring in response to paternal ethanol exposure.

	16hr-CIE		8hr-CIE	
	F1 Males	F1 Females	F1 Males	F1 Females
**Object location memory**	No change	No change	No change	No change
**Object recognition memory**	No change	No change	No change	No change
**Social Interaction**	No change	No change	No change	No change
**Acute functional tolerance**	Not performed	Not performed	Shorter recovery time after 2nd EtOH injection	No change
**Loss of righting response (LORR) (midazolam)**	Shorter LORR duration	Shorter LORR duration	Longer LORR duration	No change
**LORR (ketamine and ethanol)**	No change	No change	No change	No change
**Stress-induced hyperthermia**	Not performed	Not performed	Reduced SIH response	No change
**Drinking in the dark**	No change	Reduced consumption	No change	No change
**Every-other-day two-bottle choice**	No change	No change	No change	No change
**Marble burying test**	No change	No change	No change	No change
**Novelty suppressed feeding test**	No change	No change	No change	No change
**Home cage activity**	No change	No change	No change	No change
**Prepulse inhibition of acoustic startle**	No change	No change	No change	No change
**Bottle-brush test**	No change	No change	No change	No change
**Blood glucose levels**	No change	No change	No change	No change

## Supplementary Materials

The following are available online at https://www.mdpi.com/2076-3425/10/9/658/s1, Figure S1: Marble burying test performed on offspring from 16hr-CIE experiment. Figure S2: Novelty suppressed feeding assay performed on offspring from 16hr-CIE experiment. Figure S3: Home-cage activity recorded in offspring from 16hr-CIE experiment. Figure S4: Acoustic startle response and pre-pulse inhibition in offspring from 16hr-CIE experiment. Figure S5: Bottle-brush test performed in offspring from 8hr-CIE experiment. Figure S6: Blood glucose levels measured in offspring from 16-hr CIE experiment.
