# Supplementary material for: Effects of Paternal Preconception Vapor Alcohol Exposure Paradigms on Behavioral Responses in Offspring
Source: Brain Sci. 2020 Sep 22;10(9):658. doi: 10.3390/brainsci10090658 (PMC7564629; doi:10.3390/brainsci10090658)
Supplement: Supplementary file 1 [file brainsci-10-00658-s001.pdf]

Supplementary File:

Materials and Methods:

Marble burying test

The test was performed as reported previously [1]. Briefly, cages (15× 10 × 8 inches) were filled with mouse bedding material. Mice were allowed to explore the cages filled with bedding for 10 minutes. After habituation, mice were placed back into the home cage. Twenty glass marbles were placed equidistant on the surface of bedding in a 4 × 5 arrangement. Mice were placed in test cage containing marbles with care not to disturb the marbles. Mice were allowed to explore the marbles for 30 minutes. The number of marbles buried was hand-scored independently by two observers. A marble was scored as buried if it was covered two-thirds of its surface area by bedding.

Novelty suppressed feeding test

This test was conducted as previously described [2], with minor modifications. Mice were weighed and then food-deprived for 24 hours before the test. Testing was conducted in two different phases: initially in experimental arena and then in home cage. The experimental arena consisted of a brightly lit (325 lux) sound-enclosure open field chamber (17 × 17 × 12 inches). One food pellet was placed in lid of P60 petri dish in center of arena. The mouse was placed in left corner of arena, and the time taken to eat the food pellet was recorded. "Eating" meant an active hold and chewing on the pellet; moving the pellet or sniffing it was not scored as eating. Mouse was left in arena for 10 minutes total, then placed in a clean housing cage on rack with water and one pre-weighed pellet in center of cage (on floor with bedding). Mouse was placed back in home cage with food and water. Pellet from arena and housing cage were collected and weighed.

Homecage Activity

Mice were placed in microisolator cages inside a photobeam monitoring system (Kinder Scientific) for 3 days with ad libitum access to food and water. The locomotor activity, fine movements and basic movements were automatically recorded with the software attached with the system.

Pre-pulse Inhibition of Acoustic Startle/PPI

Mice were acclimated to the acoustic startle reflex apparatus (Kinder Scientific) holder within the attenuation chamber for 5 minutes with a constant 65 dB white noise as background noise on the day before testing. The program on the test day incorporated six trials each of six different acoustic startle pulses (65-115dB) and ten trials each of four different pre-pulse tones (70, 75, 80, and 85dB) paired with a 115dB pulse and interstimulus interval of 30msec in a pseudo-randomized order. Intertrial intervals ranged from 10-20 seconds. PPI was calculated as a percentage score: PPI (%) = (startle response for 115dB pulse alone – startle response for pre-pulse + 115dB pulse)/startle response for 115dB pulse alone × 100.

Bottle-Brush Test

This test was conducted as previously described [2]. Briefly, the mice were single-housed and testing was performed under red lights two hours after the start of the dark cycle. In this test, the mouse was attacked in the home cage with the help of a bottle brush (4.4 × 2 × 15.5 inches) moving toward it. Total 10 attacks were carried out with approx. 15-second intervals between each attack. The various responses to the attacks and number of occurrences for each response were observed.

Blood glucose levels

Offspring generated from the 16hr-CIE experiment were tested for blood glucose levels which is an essential parameter in the study of metabolism and diabetes. Mice were fasted overnight for 16 hours and blood glucose levels were measured using ONETOUCH ultra test strips (Lifescan, Switzerland) from blood drawn from the tail vein.

Results

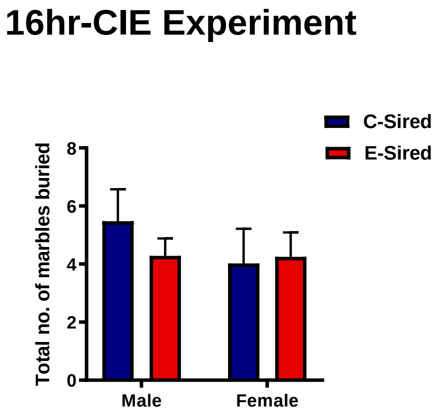

Figure S1. Marble burying test performed on offspring from 16hr-CIE experiment. There was no change in the number of marbles buried between treatment groups. Data presented as mean ± SEM (n = 16-20).

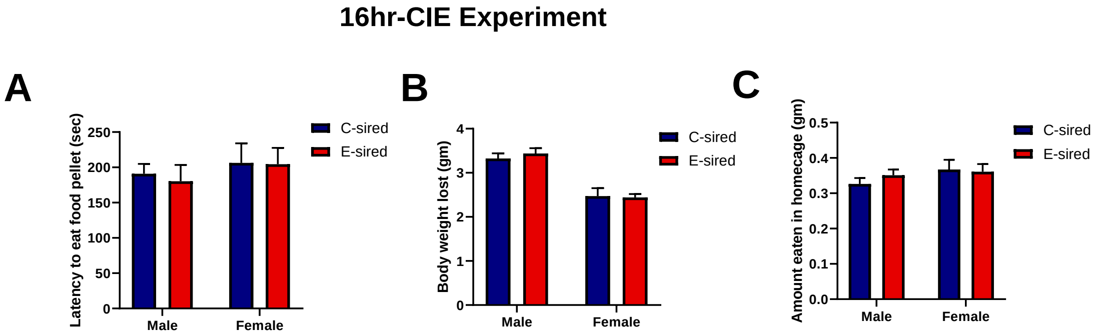

Figure S2. Novelty suppressed feeding assay performed on offspring from 16hr-CIE experiment. There was no change observed in (**A**) Latency to eat food pellet in novel environment, **(B)** Body weight lost during food deprivation period, **(C)** Amount of food eaten in the home cage between treatment groups. Data presented as mean ± SEM (n = 15-16).

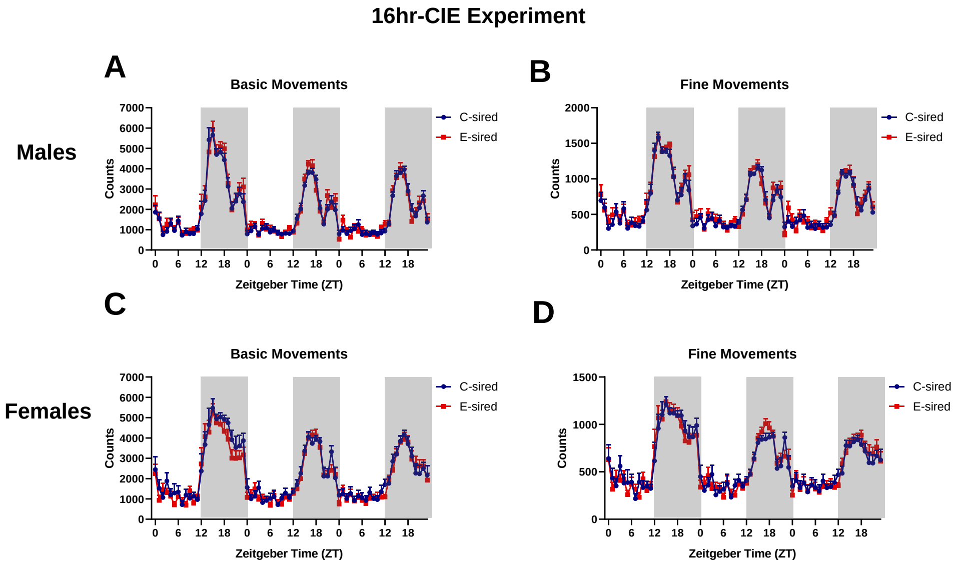

Figure S3. Home-cage activity recorded in offspring from 16hr-CIE experiment. There was no change observed in any parameters between treatment groups. **(A)** Basic movements recorded over 3 days in male offspring. **(B)** Fine movements recorded over 3 days in male offspring. **(C)** Basic movements recorded over 3 days in female offspring. **(D)** Fine movements recorded over 3 days in female offspring. Data presented as mean ± SEM (n = 16 in each group).

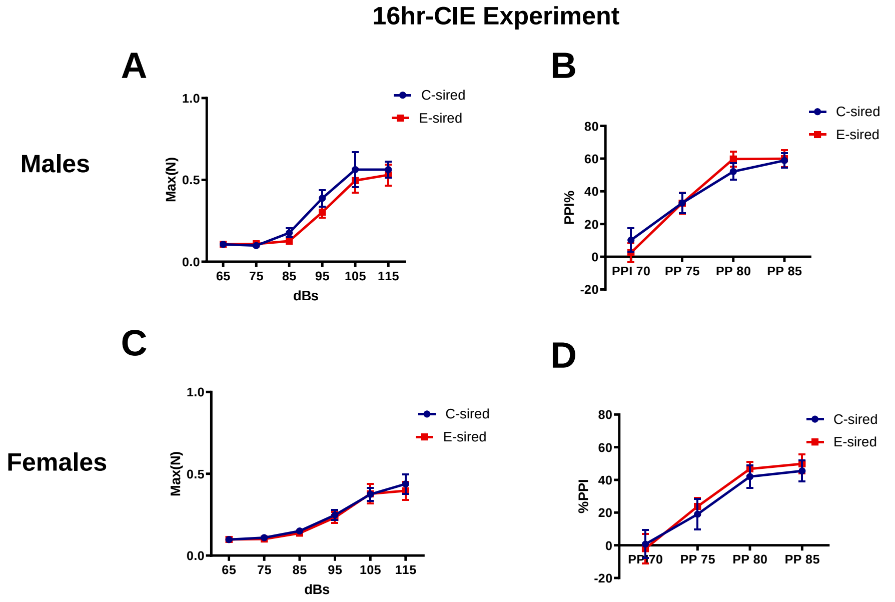

Figure S4. Acoustic startle response and pre-pulse inhibition in offspring from 16hr-CIE experiment. **(A)** Acoustic startle response in male offspring. **(B)** Pre-pulse inhibition in male offspring. **(C)** Acoustic startle response in female offspring. **(D)** Pre-pulse inhibition in female offspring. Data presented as mean ± SEM (n =16 in each group for males; n = 13 for females).

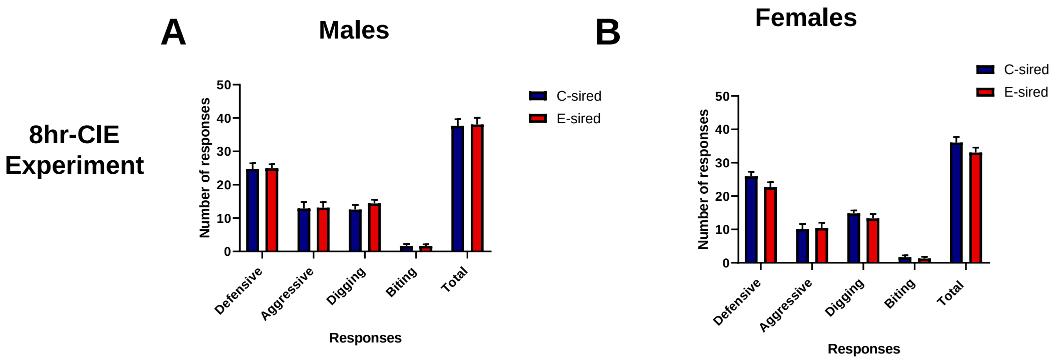

Figure S5. Bottle-brush test performed in offspring from 8hr-CIE experiment. Different behavioral responses observed in response to attack by moving a bottle brush toward **(A)** male mice **(B)** female mice. Data presented as mean ± SEM (n = 16).

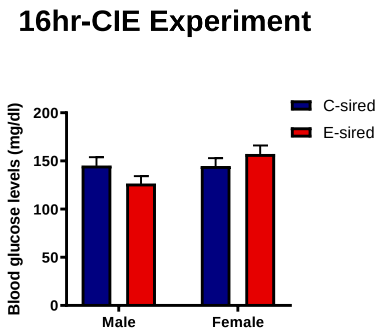

Figure S6. Blood glucose levels measured in offspring from 16-hr CIE experiment. Data presented as mean ± SEM (n = 16 in each group).

1. Eissa, N.; Jayaprakash, P.; Azimullah, S.; Ojha, S.K.; Al-Houqani, M.; Jalal, F.Y.; Lazewska, D.; Kiec-Kononowicz, K.; Sadek, B. The histamine H3R antagonist DL77 attenuates autistic behaviors in a prenatal valproic acid-induced mouse model of autism. *Sci. Rep.* **2018**, *8*, 13077.
2. Sidhu, H.; Kreifeldt, M.; Contet, C. Affective disturbances during withdrawal from chronic intermittent ethanol inhalation in C57BL/6J and DBA/2J male mice. *Alcohol. Clin. Exp. Res.* **2018**, *42*, 1281–1290.
